# Multiple roads lead to Rome: unique morphology and chemistry of endospores, exospores, myxospores, cysts and akinetes in bacteria

**DOI:** 10.1099/mic.0.001299

**Published:** 2023-02-20

**Authors:** Andrea Corona Ramírez, Kang Soo Lee, Adolfo Odriozola, Marek Kaminek, Roman Stocker, Benoît Zuber, Pilar Junier

**Affiliations:** ^1^​ Laboratory of Microbiology, Institute of Biology, University of Neuchatel, Neuchatel, Switzerland; ^2^​ Department of Civil, Institute for Environmental Engineering, Environmental and Geomatic Engineering, ETH Zurich, Zurich, Switzerland; ^3^​ Institute of Anatomy, University of Bern, Bern, Switzerland

**Keywords:** akinete, calcium dipicolinic acid (CaDPA), CEMOVIS, cyst, endospore, exospore, myxospore, Raman microspectroscopy

## Abstract

The production of specialized resting cells is a remarkable survival strategy developed by many organisms to withstand unfavourable environmental factors such as nutrient depletion or other changes in abiotic and/or biotic conditions. Five bacterial taxa are recognized to form specialized resting cells: Firmicutes, forming endospores*;* Actinobacteria*,* forming exospores*;* Cyanobacteria*,* forming akinetes; the δ-Proteobacterial order Myxococcales, forming myxospores; and Azotobacteraceae, forming cysts. All these specialized resting cells are characterized by low-to-absent metabolic activity and higher resistance to environmental stress (desiccation, heat, starvation, etc.) when compared to vegetative cells. Given their similarity in function, we tested the potential existence of a universal morpho-chemical marker for identifying these specialized resting cells. After the production of endospores, exospores, akinetes and cysts in model organisms, we performed the first cross-species morphological and chemical comparison of bacterial sporulation. Cryo-electron microscopy of vitreous sections (CEMOVIS) was used to describe near-native morphology of the resting cells in comparison to the morphology of their respective vegetative cells. Resting cells shared a thicker cell envelope as their only common morphological feature. The chemical composition of the different specialized resting cells at the single-cell level was investigated using confocal Raman microspectroscopy. Our results show that the different specialized cells do not share a common chemical signature, but rather each group has a unique signature with a variable conservation of the signature of the vegetative cells. Additionally, we present the validation of Raman signatures associated with calcium dipicolinic acid (CaDPA) and their variation across individual cells to develop specific sorting thresholds for the isolation of endospores. This provides a proof of concept of the feasibility of isolating bacterial spores using a Raman-activated cell-sorting platform. This cross-species comparison and the current knowledge of genetic pathways inducing the formation of the resting cells highlights the complexity of this convergent evolutionary strategy promoting bacterial survival.

## Data Summary

The authors confirm all supporting data, code and protocols have been provided within the article or through supplementary data files.

**Impact Statement**

All organisms strive to survive and bacteria are no exception. Some bacteria produce specialized resting cells exhibiting minimal metabolic activity, called spores, to survive unfavourable environmental conditions, such as nutrient depletion and desiccation, among others. Even though bacterial spores differ in their shape and mechanism of production, the term ‘spore’ is often associated with endospores (the best studied bacterial spore), and thus the characteristics expected from a newly discovered bacterial spore are those of endospores. However, here we were able to show through cryo-EM of vitreous sections and single-cell Raman microspectroscopy that endospores, exospores, myxospores, akinetes and cysts do not share morpho-chemical features. Hence, it is conceivable that other bacterial spore-like cells will differ in morphology, chemical composition and mechanism of formation from those in known spore-formers. Additionally, we present a proof of concept on how individual Raman microspectroscopic signatures could be used for the isolation of spores from environmental samples.

## Introduction

Micro-organisms are exposed to periodic cycles of nutrient exhaustion and different abiotic and biotic stresses that are unfavourable for growth and reproduction [1, 2]. An important survival strategy for many bacteria under these conditions is the transition into dormancy [3, 4]. There are two main strategies to transition into dormancy: the production of long-lived dormant persister cells that are resistant to antibiotics [5], and the production of specialized resistant cells called spores or cysts. The taxa recognized for the formation of specialized resistant cells are: Firmicutes, Actinomycetes and Cyanobacteria, and the Proteobacterial orders Myxococcales and Azotobacteracea. The best studied specialized structures are dormant long-lived endospores produced by members of the Firmicutes. Firmicutes correspond mostly to Gram-positive bacteria, with the exception of the Gram-negative Negativicutes and the Halanaerobiales [6, 7]. Endospores are one of the most resistant specialized dormant cells, being able to resist high temperature (up to 100 °C), ionizing radiation, chemical solvents and detergents. Endospores can remain dormant in the environment for long periods of time [8, 9]. In *Bacillus* spp., endosporulation is induced by starvation and has the engulfment of the pre-spore (smaller cell in an asymmetric cell division) by the mother cell as a hallmark (Fig. S1A, available in the online version of this article) [10, 11]. After endospores, the second most studied type of spore are exospores, which are produced by members of the phylum Actinobacteria. The production of exospores is also triggered by nutrient depletion [12, 13]. During sporulation non-branching aerial hyphae emerge from the colony surface. Then synchronous cell division is initiated to produce many identical spores that are finally released into the environment (Fig. S1B). Mature exospores produced by *Streptomyces* are more resistant to desiccation, low temperature and osmotic changes than vegetative cells [14]. However, they are less resistant to heat and desiccation than endospores [15].

In comparison to Firmicutes and Actinobacteria, *Azotobacter *, *Myxococcus* and Cyanobacteria are less studied spore-formers. In the case of *Azotobacter *, most of the research done on the physiology and morphology of cysts (specialized resistant cells) was performed in the 1970s and 1980s, while in recent years, research on *Azotobacter* has mainly focused on nitrogen fixation and its application in agriculture [16]. Thus, little is known of the encystment process and its genetic components. Cysts are resistant to desiccation and have been shown to survive in dry soil for more than 10 years [17, 18]. Under laboratory conditions, the production of cysts takes places in the late stationary phase of a culture, or it is induced by the addition of n-butanol or β-hydroxybutyrate as a carbon source (Fig. S1C) [19–21]. Like *Azotobacter *, *Myxococcus* are Gram-negative bacteria belonging to the Proteobacteria. Representatives of *Myxococcus* have a unique strategy to form resistant dormant cells called myxospores. The formation of myxospores starts when cultures reach high density and encounter starvation. The rod-shaped vegetative cells glide to aggregation centres to merge and form large mounds, which then lead to the formation of fruiting bodies. Finally, some cells inside the fruiting body will differentiate into dormant coccus-shaped myxospores, while the peripheral rod-shaped cells will survive as dormant cells (Fig. S1D) [22, 23]. Myxospores are not as resistant as endospores, but they show higher resistance to high temperature, desiccation, UV and sonication than the corresponding vegetative cells [24]. This unique multicellular strategy to form spores has been studied more than encystment, but many genetic markers and mechanisms that lead to the production of myxospores are still unknown [25–27].

Within Cyanobacteria, only two orders are known to form spore-like cells: the Nostocales and Stigonematales. They are also known for the formation of heterocysts, which are nitrogen-fixing specialized cells. The spore-like cells formed by Cyanobacteria are called akinetes and are characterized by being dormant, non-motile cells with thick walls [28, 29]. As for the other spores, nutrient depletion triggers the formation of akinetes. However, the specific triggers might be species dependent [30], which is likely the case in other sporulating taxa, as shown, for example, among different groups of Firmicutes [31]. For example, for *Anabaena variabilis,* the trigger is low light, whereas for *Nostoc punctiforme* it is phosphate starvation. Mature akinetes are bigger and have a thicker wall than the vegetative cells. In addition, one important characteristic of akinetes is that their density is higher than water, and thus they are able to sink to the bottom, where they can survive for several months or decades until conditions improve [28, 32] (Fig. S1E).

Even though the strategies used to form specialized resting cells in Firmicutes, Actinobacteria*, Azotobacter*, *Myxococcus* and Cyanobacteria differ significantly, the resulting cells all have a similar function. This has led some authors to suggest that the formation of a spore-like cell could be an ancestral characteristic in bacteria, and in particular that endosporulation could be the origin of the outer membrane in Gram-negative bacteria [33–36]. If this is the case, extant spore-like cells might still share similar features. More importantly, the key role that resistant cells (hereafter referred to as ‘spores’) play in the resilience of microbial communities [37] is a strong motivation for the evaluation of the existence of a universal marker for the identification and isolation of spores.

Therefore, in this paper we present the results of a systematic comparison of the morphology and chemical signatures of vegetative cells and spores of representatives of the five spore types presented above.

The morphological analysis was performed using optical microscopy and cryo-electron microscopy of vitreous sections (CEMOVIS [38]). The principal advantage of CEMOVIS is that it circumvents the artefacts associated with chemical fixation and sample dehydration in traditional electron microscopy sample preparation. Despite the fact that sectioning can cause its own mechanical artefacts, these are known and can be taken into account during image analysis [39, 40]. CEMOVIS provides a better understanding of the biological structure within a cell, and crucially, it has been used previously to demonstrate the unique morphological features of the cell envelope (comprising the cell membrane and cell wall) in groups such as mycobacteria [41]. This approach has been used to characterize some spores [42–44], but its more generalized use offers enormous potential for analysis of the morphology of spores.

In addition, we assessed the chemical composition of the vegetative cells and spores using single-cell confocal Raman microspectroscopy. This method provides a non-destructive, label-free, accurate and high-throughput approach that allows analysis of the functional heterogeneity of cell populations, including those of uncultured bacteria, in their natural habitat [45]. This single-cell approach overcomes the limitation of asynchronous sporulation of a bacterial population by assessing the chemical composition of individual cells (vegetative or spore). Our results show that although spores of the different model bacteria presented a thicker cell envelope, no other morphological characteristic could be identified as universal for sporulation. Similar results were found for the chemical composition. No shared chemical markers could be identified among different spore types, however specific markers were found to discriminate between vegetative cells and spores from the same genus/spore type. Additionally, as a proof of concept, we validated an endospore-specific chemical marker that could be used for the sorting and isolation of endospores from environmental samples.

## Methods

### Bacterial strains

The following bacterial strains were used: for the analysis of endospores — *Bacillus subtilis* (NEU16, Neuchâtel University culture collection), *Bacillus sphaericus* (NEU1003, Neuchâtel University culture collection), *Bacillus thuringiensis* (NEU1070/DSM350); exospores — *Streptomyces violaceoruber* (NEU1225/DSM 40783), *Streptomyces avermitilis* (NEU1226/DSM46492); myxospores — *Myxococcus xanthus* (Serengeti 01, Chinhaya 20 and Indiana MC3.5.9C15, culture collection from the Department of Environmental Systems Science at ETHZ); cysts — *Azotobacter chroococcum* (NEU1159/DSM2289); akinetes — *Anabaena cylindrica* (PCC7122) (Table 1).

**Table 1.** Bacterial strains used for each analysis and the culture conditions used for the production of vegetative and spore cells. Analysis: microscopy (M), cyo-electron microscopy of vitreous sections (CM), Raman microspectroscopy (R)

Strain	Analysis	Vegetative cells			Spores			
		Media	Temp. (°C)	Time (h)	Type	Media	Temp. (°C)	Time (days)
* Bacillus subtilis* (*NEU16*)	M, CM, R	Nutrient broth	30	12–18	Endospore	Nutrient agar	30	3
* Bacillus thuringiensis* (*NEU1070/DSM350*)	R	Nutrient broth	30	12–18	Endospore	Nutrient agar	30	3
* Bacillus sphaericus* (*NEU 1003*)	R	Nutrient broth	30	12–18	Endospore	Nutrient agar	30	3
* Streptomyces avermitilis* (*NEU1226/DSM46492*)	R	Nutrient broth	30	12–18	Exospore	Nutrient agar	30	3–5
*Streptomyces violaceoruber (NEU1225/DSM 40783*)	M, CM, R	Nutrient broth	30	12–18	Exospore	Nutrient agar	30	3–5
* Myxococcus xanthus* (*Serengeti 01*)	M, CM, R	Liquid CTT	30	12–18	Myxospore	¼ CTT (1.5 % agar)	30	3–5
* Myxococcus xanthus* (*Chinhaya 20*)	R	Liquid CTT	30	12–18	Myxospore	¼ CTT (1.5 % agar)	30	3–5
* Myxococcus xanthus* (*Indiana MC3.5.9C15*)	R	Liquid CTT	30	12–18	Myxospore	¼ CTT (1.5 % agar)	30	3–5
* Azotobacter chroococcum* (*NEU1159/DSM2289*	M, CM, R	Burk’s media	30	24	Cysts	Burk’s media sucrose+0.2 % n-butanol	30	3–5
* Anabaena cylindrica* (*PCC7122*)	M, CM, R	BG11 N	RT	36	Akinete	BG11 N	4 in the dark	10–14
								

### Sample preparation

#### Vegetative cell cultures

Vegetative cells were prepared as follows: *B. subtilis *, *B. sphaericus *, *B. thuringiensis *, *S. violaceoruber* and *S. avermitilis* were cultured in 15 ml of sterile nutrient broth (NB) (art. no. AE92.2, Carl Roth, Karlsruhe, Germany) under shaking at 120 r.p.m. at 30 °C and were collected after 12–18 h of incubation. *A. chroococcum* was cultured in 15 ml of sterile Burk’s media [46] under shaking at 120 r.p.m. at 30 °C and was collected after 24 h of incubation. The strains of *M. xanthus* were cultured in 15 ml of liquid Casitone–Tris complex medium (CTT) [47] under shaking at 120 r.p.m. at 30 °C and were collected after 12–18 h of incubation. *A. cylindrica* was cultured in 20 ml of sterile nitrogen-free BG11 [48] in a 16 h light and 8 h darkness cycle, with shaking at 70 r.p.m. at RT, and the cells were collected after 36 h of incubation (Table 1).

#### Spore induction and collection

The spores of *B. subtilis *, *B. sphaericus *, *B. thuringiensis *, *S. violaceoruber* and *S. avermitilis* were produced by inoculating Petri dishes of nutrient agar (NA) (art. no. AE92.2, Carl Roth, Karlsruhe, Germany) and then incubating them at 30 °C for 2–3 days for endospores and 3–5 days for exospores. The endospores of *B. subtilis *, *B. sphaericus* and *B. thuringiensis* were collected with the loop from the surface of the agar and resuspended in 2 ml of sterile 0.2 M glycerol solution. The exospores of *S. violaceoruber* and *S. avermitilis* were collected by adding 100 sterile glass beads (4 mm diameter) into the surface of the culture with shaking for 30 s. To recover the exospores that adhered to the beads, 5 ml of TX buffer [0.05 M tris(hydroxymethyl)aminomethane, 0.001 % (vol/vol) Triton X-100, pH 7.3] was then added and the fluid was collected into a 15 ml Falcon tube. This process was repeated twice to maximize the collection of all spores. The buffer containing the spores was then centrifuged at 10 000 ***g*** for 10 min, and then the supernatant was removed, and the spores were resuspended in 2 ml of sterile 0.2 M glycerol solution.

The cysts of *A. chroococcum* were produced as follows. Approximately 20 ml of vegetative cells were produced as indicated above in Burk’s media. After 12 h the media were centrifuged at 3 000 ***g*** and the supernatant was discarded. The pellet was then resuspended in approximately 20 ml of Burk’s sucrose-free liquid media with 0.2 % of n-butanol and was incubated at room temperature for 5–7 days.

The myxospores from *M. xanthus* strains were produced by inoculating densely on Petri dishes with ¼ CTT (CTT media with ¼ of the original Casitone) solid medium with incubation at 30 °C for 5–7 days. ¼ CTT media was used instead of TPM media to obtain a larger number of spores. The myxospores were then collected with a loop from the agar surface and resuspended in 2 ml of sterile physiological water (0.9 % NaCl). Akinetes of *A. cylindrica* were produced by placing approximately 20 ml of a stationary culture of *A. cylindrica* covered with aluminium at 4 °C for 10–14 days (Table 1).

### Optical microscopy

All optical microscopy was performed using an Upright Leica DM4 B Microscope (Leica, Wetzlar, Germany) and the images were generated with a Leica Microscope DFC7000T Camera (Leica, Wetzlar, Germany). To obtain better images, an agar pad was used to observe the cells. For this, 1.5 % agar was prepared and approximately 750 µl of it was added onto a glass slide. While still warm, a cover slip was placed on top of the agar. Once the agar was solid, the cover slip was removed and 1 µl of the vegetative culture was pipetted on top. The spores of *B. subtilis *, *S. violaceoruber* and *M. xanthus* were collected from the agar plate and resuspended in 1 ml of physiological water (0.9 %NaCl) before microscopy, whereas for spores of *A. cylindrica *, a 1 µl drop of the culture was pipetted onto the agar pad. Furthermore, to improve the identification of the *A. chroococcum* cysts, 100 µl of the spore culture was collected in a 1.5 ml Eppendorf tube and mixed with 100 µl of ‘cyst stain’ prepared as previously described [49]. The mix was allowed to rest for 10 min before it was used for microscopy, where a 1 µl drop was pipetted onto the agar pad.

### Cryo-EM of vitreous sections (CEMOVIS)

#### Sample preparation and imaging

The vegetative cells and spores from *B. subtilis *, *S. violaceoruber *, *A. chroococcum *, *M. xanthus* and *A. cylindrica* were prepared as described above; where necessary, culture volumes were doubled or tripled to obtain a higher biomass. The samples were resuspended in phosphate-buffered saline (PBS) supplemented with 30 % dextran (D1662 from Sigma-Aldrich, St Louis, MI, USA). They were then cooled under high pressure (2000 bar) to liquid nitrogen temperature (−196 °C) within milliseconds using an EM-PACT2 machine (Leica Microsystems, Wetzlar, Germany). These conditions prevent the formation of ice crystals, which can damage cellular structures [50]. The frozen samples were then cut into thin slices (50 nm) in a cryo-ultramicrotome UC6 FC6 (Leica Microsystems, Wetzlar, Germany) and placed on a R3.5–1 holey carbon EM grid (Quantifoil, Großlöbichau, Germany). Samples prepared in this way were then loaded into a Tecnai F20 transmission electron microscope (Thermo Fisher Scientific, Waltham, MA, USA) on a cryo-holder model 626 (Gatan, Pleasanton, CA, USA) keeping the sample temperature low (−180 °C) and analysed by the low-dose method at 200 kV acceleration voltage.

#### Image analysis

The CEMOVIS images were analysed using Fiji version 2.3.0/1.53 f [51]. For each species, representative high-quality images were selected to analyse the cell envelope density profile of both vegetative and spore cells. Using the line tool, a perpendicular line with a 23 pixel width was drawn across the cell envelope to reduce noise. From this line, the density profile was plotted for each image. The width of the cell wall was measured five times in different areas of the cell for five different images to obtain an average width. In the case of the Akinetes, only one image was of sufficient quality to assess the width of the cell envelope.

### Single-cell Raman microspectroscopy

A confocal Raman microspectroscope (LabRAM HR Evolution, Horiba Scientific, France) was used. This system is based on an upright microscope (Olympus BXFM, Olympus, Shinjuku, Tokyo, Japan) that is integrated with components for Raman measurements, including a 532 nm neodymium–yttrium aluminium garnet (Nd:YAG) laser (Cobolt Samba, Hübner Photonics GmbH, Kassel, Germany); a 600 lines mm^−1^ diffraction grating (blazed at 500 nm); a 100× dry objective [Olympus MPlan N, numerical aperture (NA)=0.9]; a confocal pinhole with 100 µm; and a back-illuminated deep-depleted charge-coupled device (CCD) detector (1024×256 pixels, pixel size: 26×26 µm; Horiba Scientific).

A 2 µl drop containing a sample of either vegetative cells or spores for each strain was placed on an aluminium-coated slide (AI136; EMF Corporation, Rochester, NY, USA) and dried at 30 °C for 15 min. The sample was briefly rinsed in 0.2 M glycerol solution to remove the residual traces of the medium. The sample was then dried under a constant air flow and the Raman spectra of single cells were measured. A 10 mW laser was focused on a single cell and a spectral window of 800–3300 cm^−1^ (3.5 cm^−1^ resolution) was measured with a 10 s exposure time. For *A. cylindrica* PCC7122 that generates strong Raman signals (resonance Raman scattering due to the presence of carotenoids [52]), a 0.3 mW laser power and a 1 s exposure time were used. This additional manipulation is not expected to modify the chemical signatures obtained. For each sample 15–20 individual cells were measured, and a representative Raman spectrum was normalized with respect to the maximum and minimum intensities (*I − I*_min_ / *I*_max_ − *I*_min_) are shown. No further data processing (smoothing or baseline subtraction) was conducted.

### Validation of an endospore-specific marker

Nine endospore-forming bacteria were used to establish an endospore-specific chemical marker. These were compared with other spore-formers and asporogenic bacteria. The endospore-forming bacteria used were *B. subtilis* (NEU16, Neuchâtel University culture collection), *B. thuringiensis* (NEU1070/DSM350), *B. sphaericus* (NEU1003, Neuchâtel University culture collection), *Paenibacillus marcerans* (NEU1004), *Paenibacillus alvei* (NEU1291, Neuchâtel University culture collection), *Bacillus cereus* (SPO95, Neuchâtel University culture collection), *Bacillus weihenstephanensis* (SPO146, Neuchâtel University culture collection), *Bacillus cereus* (SPO91, Neuchâtel University culture collection) and *Bacillus* sp. (SPO122, Neuchâtel University culture collection). Other spore-forming bacteria were used for comparison. These corresponded to: *S. violaceoruber* (NEU1225/DSM 40783), *S. avermitilis* (NEU1226/DSM46492), *Actinomycetes* sp. (NEU70, Neuchâtel University culture collection), *A. chroococcum* (NEU1159/DSM2289; *A. cylindrica* (PCC7122), and the asporogenic bacteria *Arthrobacter nicotianae* (NEU1123/20123) and *Arthrobacter globiformis* (NEU1124/DSM20124). The growth conditions for each strain are presented in Table 2. The method used for the sample preparation for the vegetative cells and spores is described above.

**Table 2.** Bacterial strains used for the establishment of an endospore threshold and the culture conditions used for the production of vegetative and spore cells

Strain	Vegetative cells			Spores			
	Media	Temp. (°C)	Time (h)	Type	Media	Temp. (°C)	Time (days)
* Bacillus subtilis* (NEU16)	Nutrient broth	30	12–18	Endospore	Nutrient agar	30	3
* Bacillus thuringiensis* (NEU1070/DSM350)	Nutrient broth	30	12–18	Endospore	Nutrient agar	30	3
* Bacillus sphaericus* (NEU 1003)	Nutrient broth	30	12–18	Endospore	Nutrient agar	30	3
*Paenibacillus marcerans* (NEU1004)	Nutrient broth	30	12–18	Endospore	Nutrient agar	30	3
* Paenibacillus alvei* (NEU1291)	Nutrient broth	30	12–18	Endospore	Nutrient agar	30	3
* Bacillus cereus* (SPO95)	Nutrient broth	30	12–18	Endospore	Nutrient agar	30	3
* Bacillus weihenstephanensis* (SPO146)	Nutrient broth	30	12–18	Endospore	Nutrient agar	30	3
* Bacillus cereus* (SPO91)	Nutrient broth	30	12–18	Endospore	Nutrient agar	30	3
*Bacillus sp*. (SPO122)	Nutrient broth	30	12–18	Endospore	Nutrient agar	30	3
* Streptomyces avermitilis* (NEU1226/DSM46492)	Nutrient broth	30	12–18	Exospore	Nutrient agar	30	3–5
* Streptomyces violaceoruber* (NEU1225/DSM40783)	Nutrient broth	30	12–18	Exospore	Nutrient agar	30	3–5
*Actinomycetes sp*. (NEU70)	Nutrient broth	30	12–18	Exospore	Nutrient agar	30	3–5
* Arthrobacter globiformis* (NEU1124/DSM20124)	Nutrient broth	30	12–18	na	na	na	na
* Arthrobacter nicotianae* (NEU1123/20123)	Nutrient broth	30	12–18	na	na	na	na
* Azotobacter chroococcum* (NEU1159/DSM2289)	Burk’s media	30	24	Cysts	Burk’s media sucrose+0.2 % n-butanol	30	3–5
* Anabaena cylindrica* (PCC7122)	BG11 N	RT	36	Akinete	BG11 N	4 in the dark	10–14

NA, Not applicable.

#### Single-cell Raman microspectroscopy in liquid

In order to implement a sorting step in a microfluidics device, the cells must be analysed in liquid. Therefore, the measurements were made in liquid to make the analysis pipeline compatible with a fluid-based sorting capability. The same confocal Raman microspectroscope (LabRAM HR Evolution, Horiba Scientific, France), laser, confocal pinhole and detector as indicated above were used. In this case, the system was based on an inverted microscope (Nikon Ti-E). A 300 lines mm^−1^ diffraction grating (blazed at 600 nm) and a 63× water-immersion objective (Nikon SR Plan Apo IR 60XC WI, NA=1.27) were installed. Additionally, optical tweezers formed using a 1064 nm laser were used to immobilize cells during the Raman measurement. For these measurements, a sample of either vegetative cells or spores was resuspended in 0.2 M glycerol solution and a 5 µl drop of the sample was put in a microchamber. The sample was sandwiched between two glass coverslips (150 µm thickness) and two coverslips were placed between these coverslips as a spacer, providing the fluid microchamber with a thickness of 150 µm. Single cells were captured using optical tweezers (formed using a 1 064 nm laser) and their Raman spectra were measured. A spectral window of 400–3300 cm^−1^ was measured with a 100 mW laser power and a 1 s exposure time. For *A. cylindrica* PCC7122, a 3 mW laser power and a 1 s exposure time were used. For each sample 5 to 26 individual cells were measured.

## Results

### Morphology

**Fig. 1.**
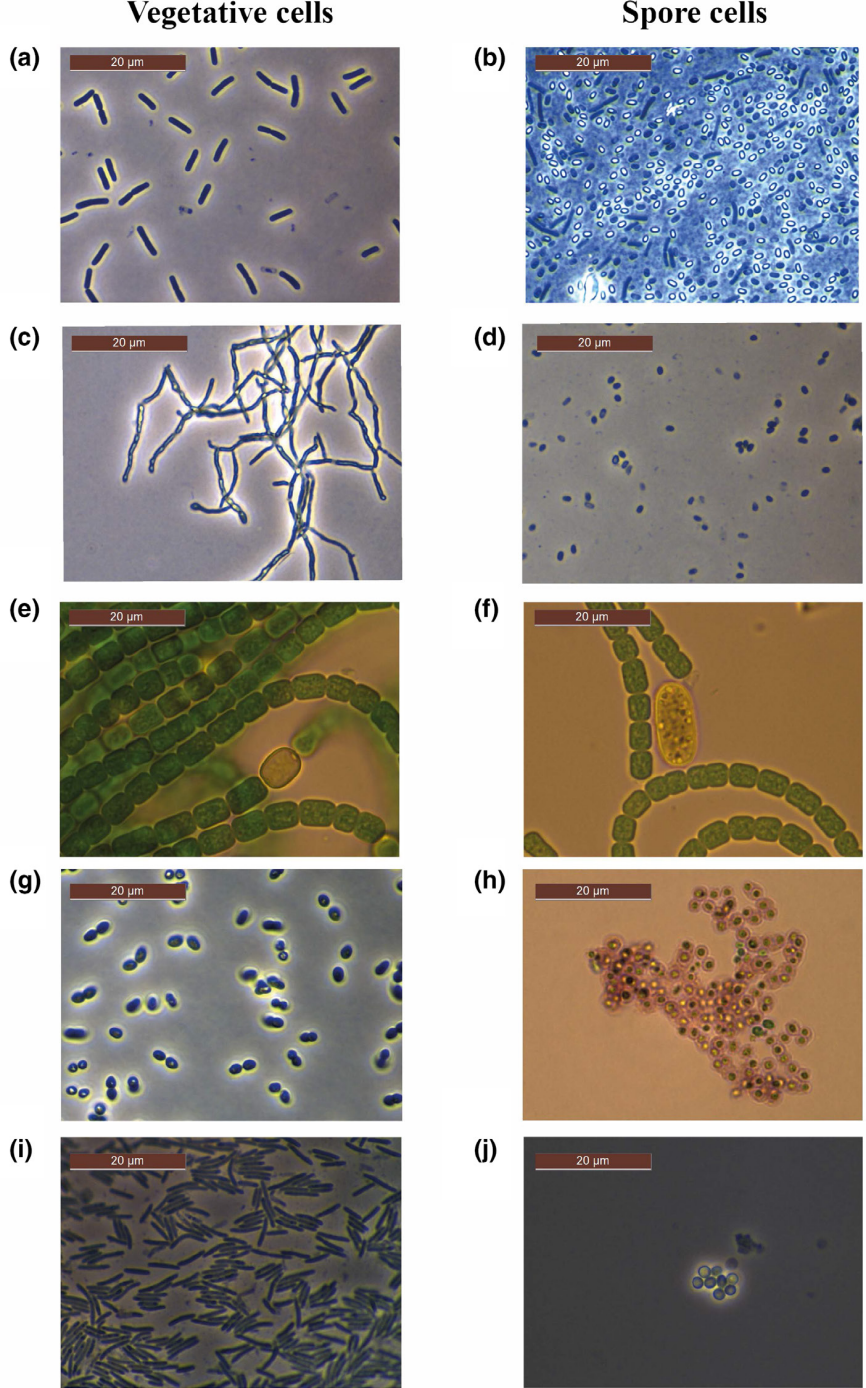
Microscopic images of vegetative cells and spores of (a) rod-shaped vegetative cells of *B. subtilis *, observed in phase contrast mode, and (**b**) phase-bright spore cells of *B. subtilis *, observed in phase contrast mode. Elongated dark cells are vegetative cells and dark oval cells are endospores out of focus. (**c**) Mycelium-like vegetative cells of *S. violaceoruber *, observed in phase contrast mode, (**d**) ovoidal mature spore cells of *S. violaceoruber *, observed in phase contrast mode, (**e**) vegetative cell chain with a round/yellow heterocyst of *A. cylindrica *, observed in brightfield mode, (**f**) big Akinete of *A. cylindrica *, surrounded by a chain of vegetative cells, observed in brightfield mode, (**g**) vegetative cells of *A. chroococcum *, observed in phase contrast mode, (**h**) mature cysts of *A. chroococcum *, stained with ‘cyst stain’ and observed in brightfield mode, (**i**) elongated rod-shaped vegetative cells of *M. xanthus *, observed in brightfield mode, (**j**) light refracting, mature spore cells of *M. xanthus *, observed in brightfield mode. The bar on the left top corner indicates the scale.

The vegetative cells of the model micro-organisms underwent a very conspicuous morphological transformation during the formation of spores (Fig. 1). The spores belonging to *B. subtilis *, *S. violaceoruber *, *A. chroococcum* and *M. xanthus* showed a significant reduction in cellular size (Fig. 1b, d, h, j) and rounding up of the cells, while the opposite was observed in spores of *A. cylindrica* (Fig. 1f). The spores of *A. cylindrica* were approximately three times larger than the vegetative cells. Furthermore, when observed in phase contrast mode, the spores of *B. subtilis* and *M. xanthus* refracted light (Fig. 1b, j). This light refracting characteristic could also be observed in the spores and vegetative cells of *A. cylindrica* (data not shown), whereas no light refraction was observed in the spores of *S. violaceoruber* and *A. chroococcum *.

**Fig. 2.**
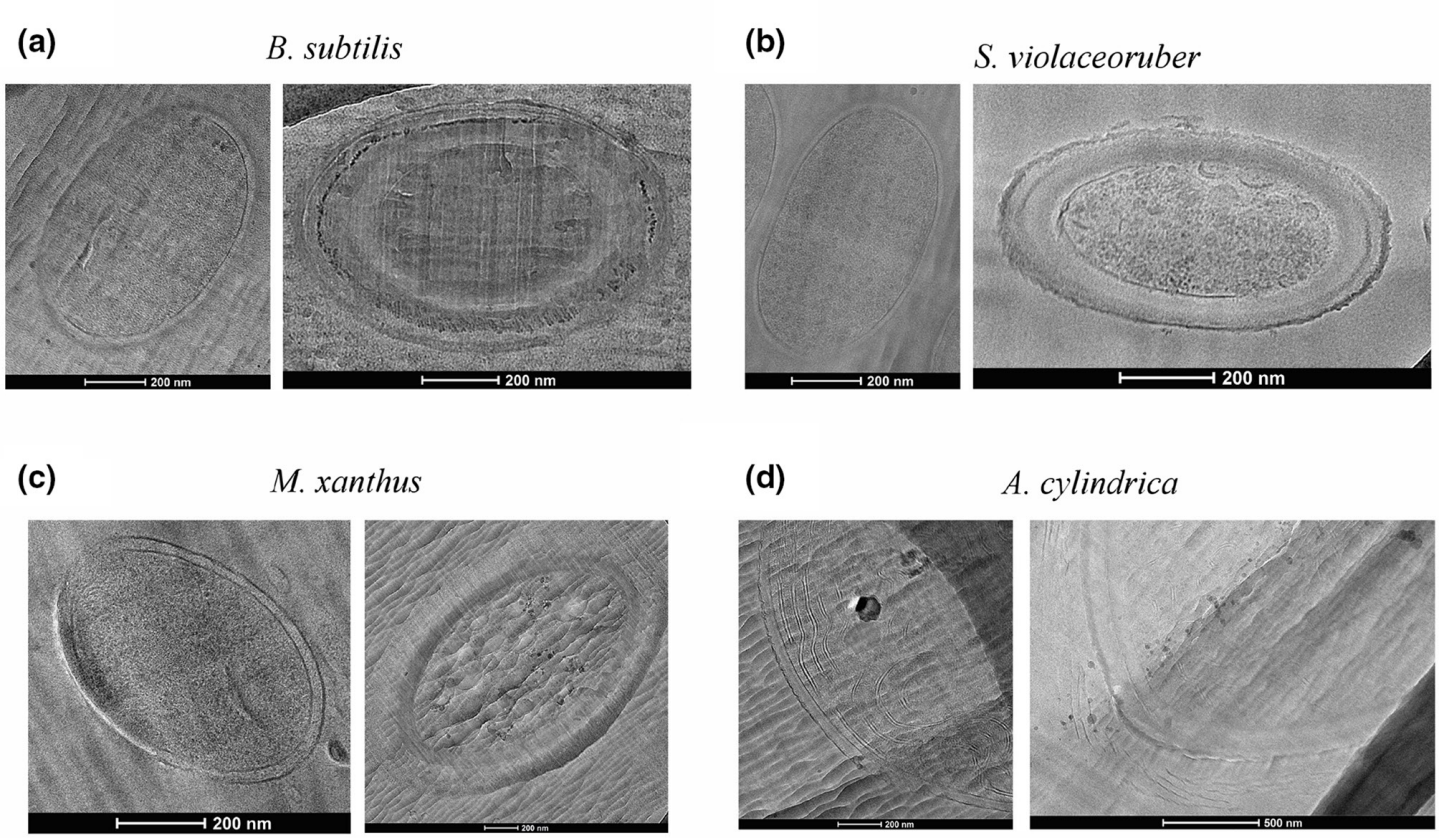
Cryo-electron microscopy of vitreous sections of vegetative cells (left side) and spores (right side) of (a) *B. subtilis,* (**b**) *S. violaceoruber *, (**c**) *M. xanthus* and (d) *A. cylindrica *. The bar at the bottom of each image indicates the scale.

Images based on cryo-EM of vitreous sections (CEMOVIS) were obtained for vegetative cells and spores from *B. subtilis *, *S. violaceoruber *, *M. xanthus* and *A. cylindrica* (Fig. 2). CEMOVIS images of *Azotobacter* spores could not be obtained due to very low spore concentration and water crystallization. Vegetative cells of *B. subtilis* and *S. violaceoruber* (Fig. 2a, b; left side) presented an inner cellular membrane, a thick peptidoglycan layer and a granulated cytoplasm. The vegetative cells of *M. xanthus* and *A. cylindrica* (Fig. 2c, d; left side) presented an inner and an outer membrane and a granulated cytoplasm and, in the case of *A. cylindrica *, the cytoplasm included curved lines, which corresponded to the thylakoid membrane. The cytoplasm of the spores of *B. subtilis *, *S. violaceoruber *, *M. xanthus* and *A. cylindrica* (Fig. 2a–d; right side) exhibited a denser and smoother aspect, and the cell envelope was thicker.

**Fig. 3.**
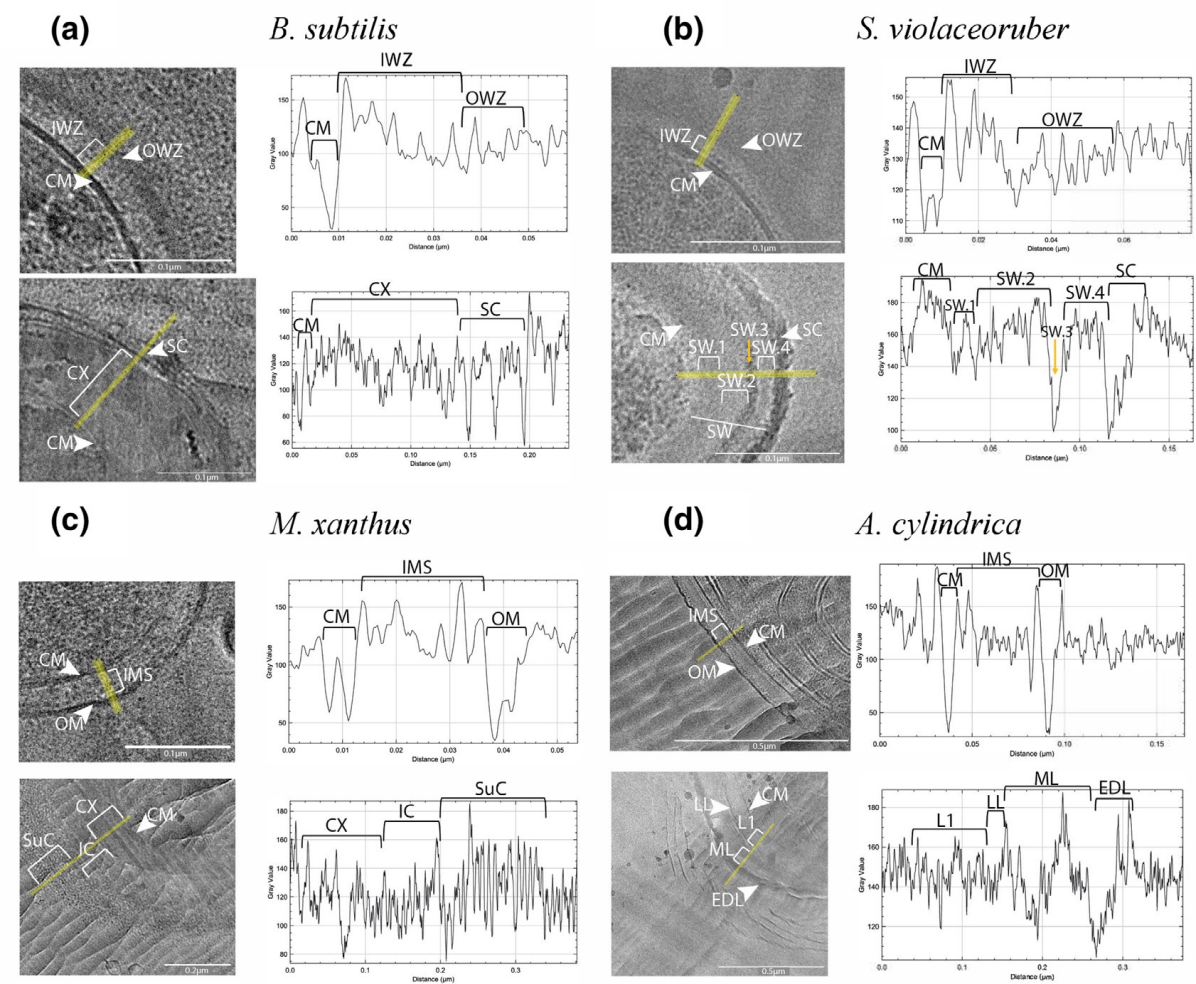
Cryo-electron microscopy of vitreous sections of the cell envelope of vegetative cells (top) and spores (bottom) and corresponding density profile of (a) *B. subtilis *, (**b**) *S. violaceoruber *, (**c**) *M. xanthus* and (d) *A. cylindrica *. In the case of (a) (* B. subtilis *), during sectioning the cell was cut along its narrower axis and therefore the final shape does not correspond to a traditional rod. The density profile was always measured perpendicular to the knife lines (except for the spores of *B. subtilis *) and corresponds to the drawn yellow line on the cell envelope. The width of the line was 23 to reduce noise. The different sections of the vegetative cells and spore’s envelopes are indicated: inner wall zone (IWZ), cortex (CX), cell membrane (CM), outer wall zone (OWZ), spore coat (SC), spore wall (SW), spore wall section 1 (SW.1), spore wall section 2 (SW.2), spore wall section 3 (SW.3), spore wall section 4 (SW.4), intermembrane spacer (IMS), outer membrane (OM), layer 1 (**L1**), mucilaginous layer (ML), laminated layer (LL) and electron-dense layer (EDL).

To further assess the changes in the cell envelope, we measured the cell envelope width (Table 3) and established a density profile. The characteristic cell envelope of Gram-positive bacteria could be observed in *B. subtilis* and *S. violaceoruber* (Fig. 3a, b; upper). The envelope was composed of the cell membrane (CM), the inner wall zone (IWZ) and an outer wall zone (OWZ) [53, 54]. In the case of *M. xanthus* and *A. cylindrica* (Fig. 3c, d; top), both Gram-negative bacteria, one could observe the CM, a thin intermembrane spacer (IMS) layer (which included the peptidoglycan and periplasm), and the outer membrane (OM).

When comparing the vegetative cell and endospore of *B. subtilis* (Fig. 3a), a few differences could be observed. Firstly, the enlargement of the cell envelope from 44.72 nm in the vegetative cell to 213.7 nm in the endospore. Second, the endospore presented two more layers outside the CM, the cortex (CX; average lower grey values than the IWZ of the vegetative cell), and a laminated spore coat (SC). In between the SC surrounding the CX, there is a second CM resulting from the engulfment process that is not always visible in the CEMOVIS images. In the case of *S. violaceoruber* (Fig. 3b) we also observed an enlargement of the cell envelope from 36.28 nm in the vegetative cell to 96.76 nm in the exospore, resulting from a thick spore wall (SW), with two different high (SW.2 and SW.4) and low grey value regions (SW.1, SW.3), followed by a low grey value region, which corresponded to the SC.

**Table 3.** Cell envelope width measurements of the different bacterial cells in vegetative (VEG) or spore (SPO) form. The average width of five measurements per image for five different cells are shown as cell 1 to cell 5. However, in the case of akinetes from *A. cylindrica *, the measurements correspond to five measurements from a single cell. The measurements are shown in nm

Bacteria	Cell type	Cell 1	Cell 2	Cell 3	Cell 4	Cell 5	Average length (nm)
* B. subtilis *	VEG	63.2	41.6	29.4	39.8	49.6	44.72
	SPO	237	180.6	225.5	209.2	216.2	213.70
* S. violaceoruber *	VEG	40	50	31	27.4	33	36.28
	SPO	97.4	67.8	92.2	107	119.4	96.76
* M. xanthus *	VEG	26.6	30	34.8	33	30.8	31.04
	SPO	249.6	246.6	272.8	177.4	269.8	243.24
* A. cylindrica *	VEG	63	50	70.6	51.6	58.4	58.72
	SPO	199	230	210	207	238	216.80

The Gram-negative cell envelope of the vegetative cells of *M. xanthus* went from 31.04 nm in width to 243.24 nm in the spores. Furthermore, the spores showed the presence of both a CM and the intermediate coat (IC) surrounding a thick CX layer. In addition, the spore also presented a surface coat (SuC) that surrounded the IC (Fig. 3c) [55]. These morphological changes could also be observed when comparing the density profile of the vegetative cell and the spore of *M. xanthus *. The density profile of the CX and IC showed higher variation in the spore than the IMS in the vegetative cell, while the SuC had a higher grey value than the OM in the vegetative cell.

The typical structure of a Gram-negative cell envelope was also detected in the vegetative cell of *A. cylindrica* (Fig. 3d). Furthermore, the akinete showed a thicker cell envelope (216.8 nm) in comparison to the vegetative cell (58.72 nm). In the akinete, outside the CM, two layers were present: layer 1 (L1) and possibly the mucilaginous layer (ML). These layers seemed to be separated by a thin laminated layer (LL), which presented a lower grey value. The most exterior layer (low grey value) has previously been denominated as electron-dense layer (EDL) [30]. Outside the EDL, several dense lines were observed surrounding the cells, although these lines were not present in other images.

### Chemical composition

**Fig. 4.**
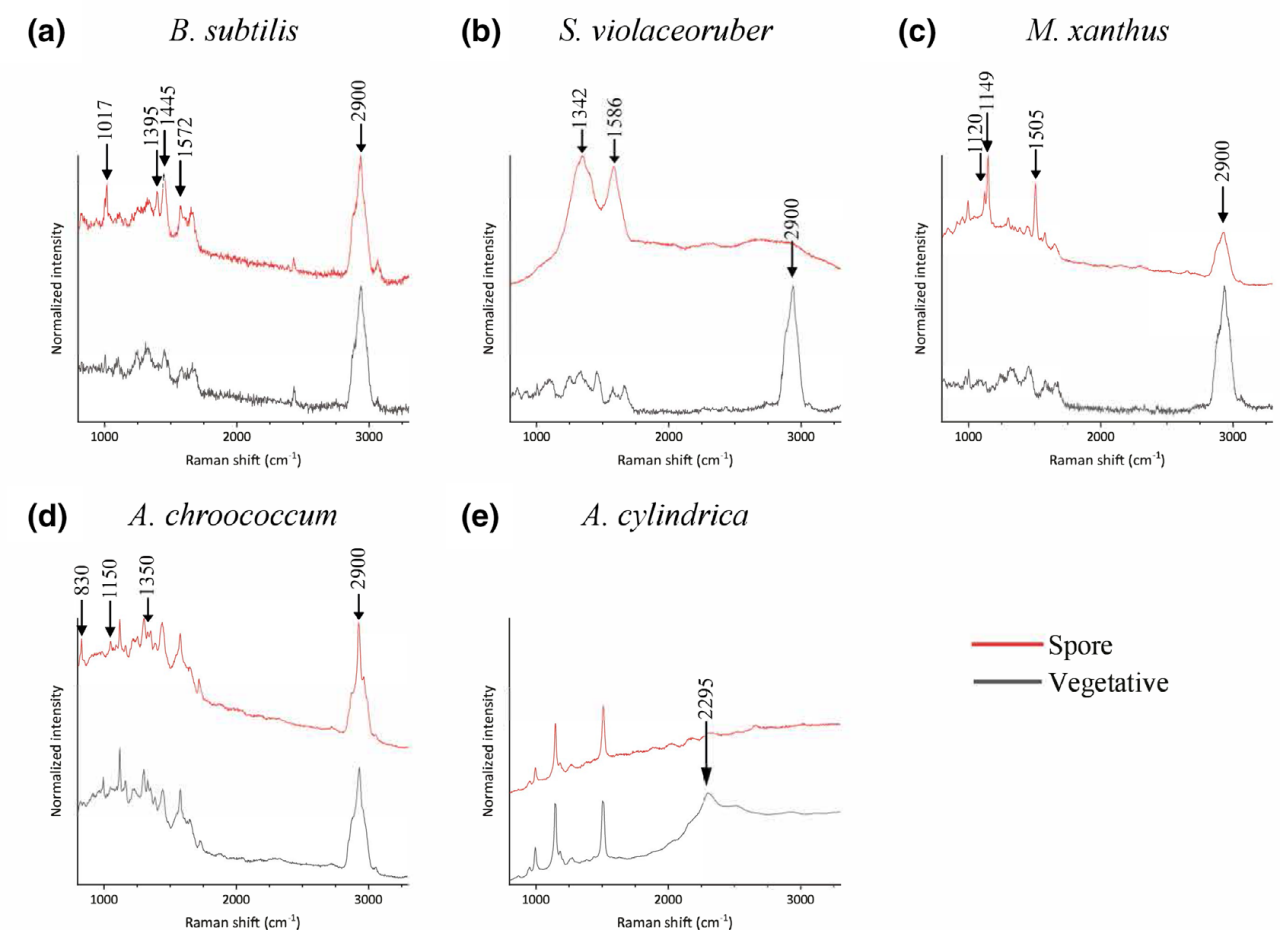
Representative Raman spectra of a vegetative cell or spore of (a) *B. subtilis *, (**b**) *S. violaceoruber *, (**c**) *M. xanthus,* (**d**) *A. chroococcum* and (e) *A. cylindrica *. The arrows indicate specific peaks that are unique to either the vegetative cell or spore; values are given in cm^−1^. For each sample 15 to 20 individual cells were measured, and a representative Raman spectrum normalized with respect to the maximum and minimum intensities (*I − I*_min_ / *I*_max_ − *I*_min_) is displayed. No further data processing (smoothing or baseline subtraction) was conducted.

We assessed the chemical composition of individual vegetative cells and spores using single-cell Raman microspectroscopy. In Fig. 4(a) representative spectra per bacteria are shown for the vegetative cells and spores of *B. subtilis *, *S. violaceoruber *, *A. chroococcum *, *M. xanthus* and *A. cylindrica* (the analysis of additional strains can be found in Fig. S2). When comparing the spectra belonging to the vegetative cells and spores of *B. subtilis* (Fig. 4a), a peak at around 2900 cm^−1^ was observed in both spectra. This peak usually corresponds to a CH_3_ or CH_2_ bond. A clear difference between the two spectra of *B. subtilis* was the intensity in the peaks belonging to calcium dipicolinic acid (CaDPA; 1017, 1395 and 1446 cm^−1^ [56]), which were only present in the spores. This chemical, CaDPA, is a well-known component of mature endospores [57, 58]. In the vegetative cell’s spectra of *S. violaceoruber* (Fig. 4b) the CH_3_/CH_2_ bond was also found, but it was not present in the spore’s spectra. Furthermore, the spore spectra of *S. violaceoruber* showed a different topography to those of the vegetative cells, presenting two main peaks, one at 1342 cm^−1^ and one at 1586 cm^−1^. The vegetative cells and spores of *M. xanthus* (Fig. 4c) presented the CH_3_/CH_2_ bond peak. Three peaks (1120; 1149 and 1550 cm^−1^) in the spore spectra had a distinctive intensity as compared to the vegetative cell. The Raman spectra of the vegetative cell and spore of *A. chroococcum* (Fig. 4d) presented a similar topography, including the CH_3_/CH_2_ bond peak. Nonetheless, the spore’s spectra presented three peaks (830, 1150 and 1350 cm^−1^) with increased intensity. Finally, the spore and vegetative spectra of *A. cylindrica* (Fig. 4e), showed the same topography, except for a peak at 2295 cm^−1^ in the vegetative spectra, which was not present in the spore spectra. Notably, both cells and spores lacked the CH_3_/CH_2_ bond peak found in all other spectra except for spores of *S. violaceoruber* (Fig. 4b).

When comparing the spore spectra of the different species*,* no conserved region was found, but with the exception of *A. cylindrica,* unique differences between the vegetative cell and its corresponding spore spectra were detected. These regions were the peaks at 1017, 1395 and 1443 cm^−1^ for endospores, the peaks at 1345 and 1586 cm^−1^ for exospores, the peaks at 1120, 1149 and 1505 cm^−1^ for myxospores, and the peaks at 830, 1150 and 1350 cm^−1^ for cysts. All these peaks were unique to each spore type (Fig. 4).

### Validation of an endospore-specific Raman marker

**Fig. 5.**
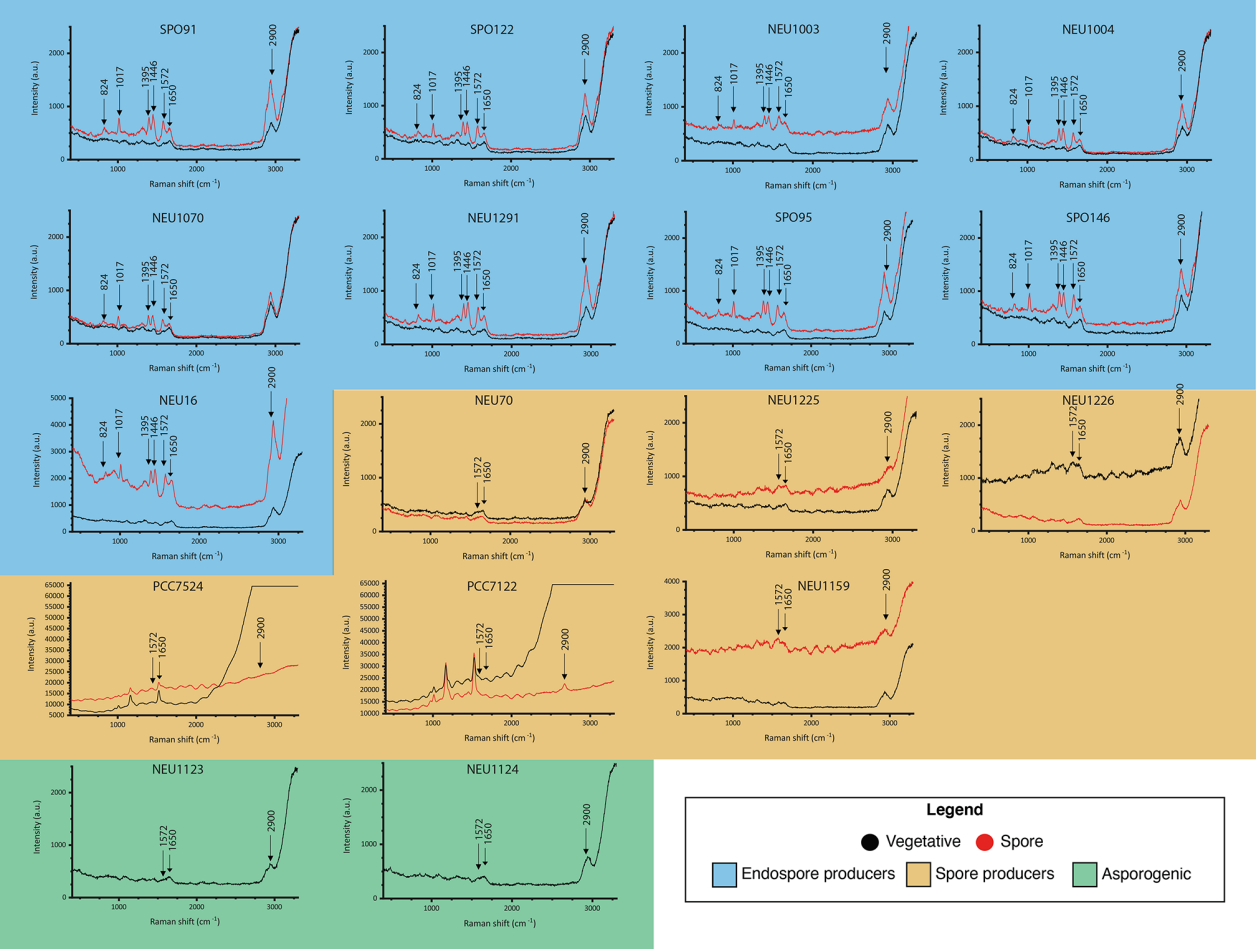
Representative Raman spectra of a vegetative cell (black) or spore (red) of endospore-forming bacteria (orange), spore-forming bacteria (blue) and asporogenic bacteria (green). The cells were suspended in a 0.2 M glycerol solution. For each sample 15 to 20 individual cells were measured, and a representative Raman spectrum normalized with respect to the maximum and minimum intensities (*I − I*_min_ / *I*_max_ − *I*_min_) is displayed. No further data processing (smoothing or baseline subtraction) was conducted.

The Raman spectra of endospores and vegetative cells from 14 different endospore-producing bacterial strains and 2 asporogenic bacterial strains were measured with the aim of evaluating the potential of the unique spore peaks (CaDPA; 1017, 1395 and 1446 cm^−1^) as endospore-specific Raman markers for cell sorting in a complex microbial community. The measurements were conducted in liquid to make the pipeline compatible with a fluid-based sorting capability. The average Raman spectra of the vegetative cells and spores for each bacterium are presented in Fig. 5. The peak at 2900 (CH_3_/CH_2_ bond) cm^−1^ was confirmed to be present in all spectra (Fig. 5). Furthermore, all of the vegetative cells’ spectra presented a similar flattened topography, while the spores’ spectra presented pronounced peaks. In the endospore spectra, characteristic CaDPA peaks were observed at 1017, 1395 and 1446 cm^−1^. None of these peaks were found in either the vegetative cells, exospores, myxospores, cysts or akinetes. Although the CaDPA peaks were present in all of endospores’ spectra, their intensity varied between the different spores, as well as between individual cells. This is clearly exemplified in Fig. 5, showing that the intensity of the spore’s spectra in *B. subtilis* (NEU16) was more than three times higher than that in *B. thuringiensis* (NEU1070). Moreover, in *B. thuringiensis* (NEU1070) and *P. marcerans* (NEU1004) (Fig. 5, respectively)*,* both the vegetative and the spore spectra presented similar intensities, except for the CaDPA peaks, which showed an increase in intensity in the spore’s spectra.

**Fig. 6.**
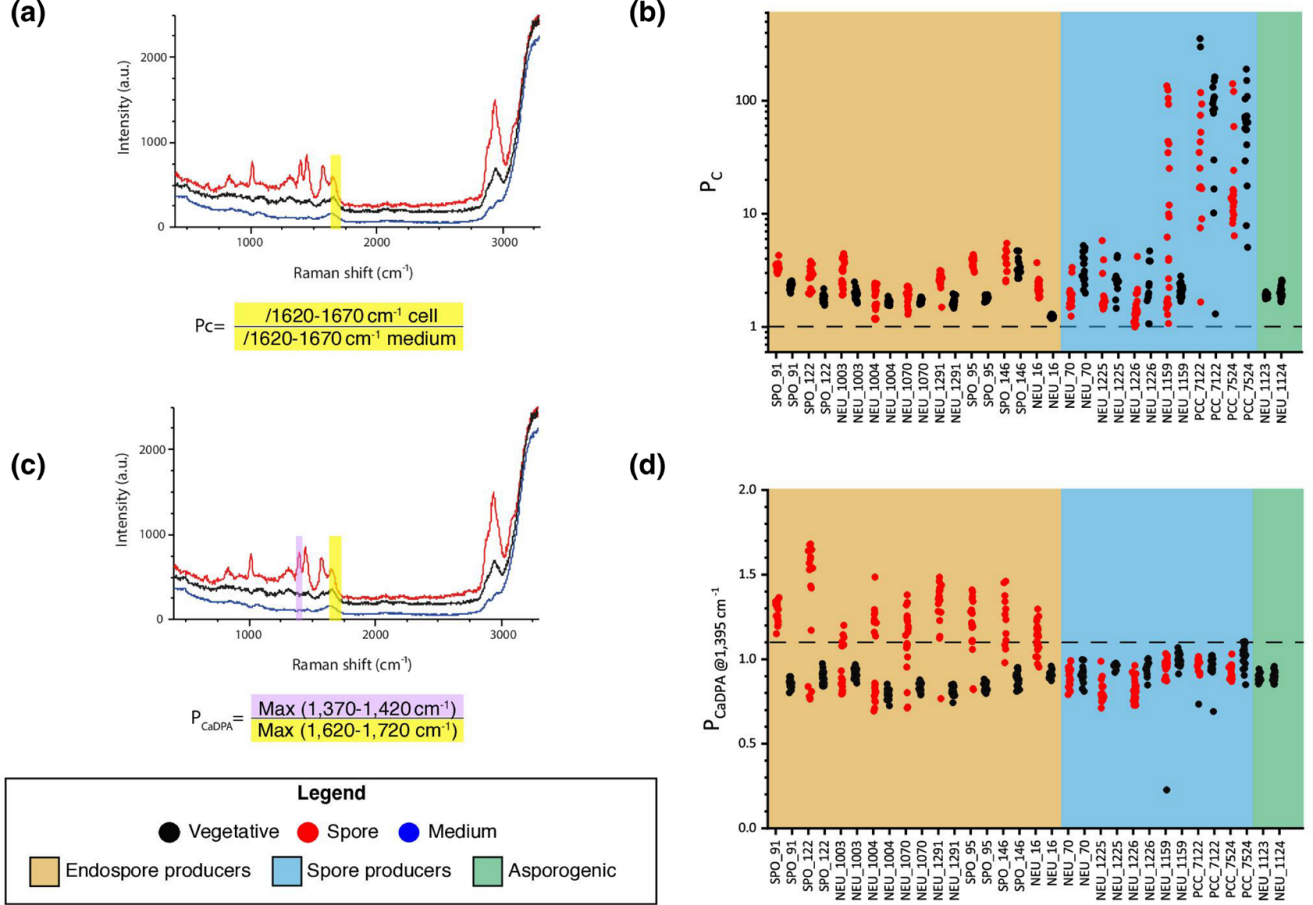
Establishment of the endospore-specific Raman marker. (**a**) Standardized Raman spectra of a representative endospore-forming bacteria (SPO91—* B. cereus *). The spore is shown in red, while the vegetative cell is shown in black. The background [corresponding to the medium (0.2 M glycerol)] is shown in blue. The region from which cell threshold (Pc) was calculated (1620 to 1670 cm^−1^) is highlighted in yellow. The formula used to calculate the Pc is given underneath the plot. This value was calculated by dividing the integrated intensity of the 1620 to 1670 cm^−1^ region of the measured cell (vegetative cell, spore, or debris) by the integrated intensity of the 1620 to 1670 cm^−1^ region of the medium. (**b**) Plot of the Pc values (*y*-axis) from individual vegetative cells (black) and spores (red) of endospore-forming bacteria (blue), spore-forming bacteria (orange) and asporogenic bacteria (green). Strain IDs are indicated on the *x*-axis, with the dashed line indicating established cell threshold. (**c**) Standardized Raman spectra of a representative endospore-forming bacteria (SPO91 *B. cereus *). The spore is shown in red, while the vegetative cell is shown in black. The background [corresponding to the medium (0.2 M glycerol)] is shown in blue. The two regions from which endospore threshold (P_CaDPA_) was calculated (1370 to 1420 cm^−1^ and 1620 to 1720 cm^−1^) are highlighted in purple and pink, respectively. The formula used to calculate the P_CaDPA_ is given underneath the plot. This value was by dividing the maximum intensity of the 1370 to 1420 cm^−1^ region of the measured cell (vegetative cell or spore) by the maximum intensity of the 1620 to 1670 cm^−1^ region of the measured cell. (**d**) Plot of the P_CaDPA_ values (*y*-axis) from individual vegetative cells (black) and spores (red) of endospore-forming bacteria (blue), spore-forming bacteria (orange) and asporogenic bacteria (green). Strain IDs are indicated on the *x*-axis, with the dashed line indicating established cell threshold.

The single-cell Raman sorting platform requires an established threshold to first discriminate bacterial cells (Pc) from other debris in the environmental samples, and a second threshold to identify endospores from vegetative cells (P_CaDPA_). To discriminate cells from debris, the peaks at 1650 cm^−1^ and 2900 cm^−1^ (CH_3_/CH_2_ bond) were initially considered. However, the 2900 cm^−1^ peak is highly variable between different cells and, as it is located at the end of the spectra, is more susceptible to being altered by background noise. For these reasons, the cell discriminating threshold was established using the peak at 1650 cm^−1^ (the region from 1620 to 1670 cm^−1^) (Fig. 6a). Even though this region does not correspond to a specific molecular bond, it was specific for cells and did not present high variation depending on experimental conditions. The ratio between the peak of the cell and that of the fluid (integrated intensity at 1620 to 1670 cm^−1^) was calculated for each measurement. Once the ratio was calculated for each cell, the cell threshold (Pc) value was established at one, to capture as many cells as possible (Fig. 6b). Any particle or cell with a ratio lower than one would be directly sorted to waste, while any cell with a ratio value higher than one would be further analysed to identify whether the cell corresponded to an endospore or a vegetative cell. The endospore threshold was established by calculating the ratio between the integrated intensity of the CaDPA peak at 1395 cm^−1^ (CaDPA) and that of the ‘cell peak’ at ~1650 cm^−1^ (Fig. 6c). This ratio was below one for all vegetative cells, as the CaDPA peak is absent. For endospores the ratio was higher than one given that the CaDPA peak presented higher intensity than the peak at 1650 cm^−1^ (Fig. 6d). As a conservative measure to ensure the isolation of endospores only, the endospore threshold (P_CaDPA_) was established at 1.1. Based on the thresholds validated here it is expected that any cell with a ratio lower than 1.1 will be directly sorted to the vegetative compartment, while any cell with a ratio value higher than 1.1 will be sorted to the endospore compartment.

## Discussion

Even though the production of the different types of spores (endospores, exospores, cysts, myxospores and akinetes) follows distinctive pathways, all of them have a common function: promoting long-term survival. This allows organisms to adapt to changes in their individual environmental niches and survive in time and/or space until they encounter conditions acceptable for vegetative growth [59]. The importance of this ecological function and its relevance, for instance, for the forecasting of resilience of microbial communities to environmental changes [4] provides a strong incentive to attempt the identification of universal markers to study sporulation beyond the models of endospores and exospores [15]. To this end, here the morphological characteristics of the model sporulating bacteria *B. subtilis *, *S. violaceoruber *, *M. xanthus, A. cylindrica* and *A. chroococcum* were assessed and compared by optical microscopy, CEMOVIS and Raman microspectroscopy.

When comparing the vegetative cells and spores produced by these organisms, the only shared characteristic that could be identified was the enlargement of the cell envelope in spores. Although the reduction in cell size is characteristic of endospores, exospores, myxospores and cysts, this was not the case for akinetes, in which enlargement is a recognized feature [28, 60]. While the CEMOVIS images revealed significant structural changes in the cell envelope, common structural components in all spore types were absent.

Similarly, the chemical analysis by single-cell Raman microspectroscopy showed that no conserved chemical marker is shared among the different spores compared in this study. Instead, unique differences between the vegetative cell and its corresponding spore spectra were detected. The two peaks observed in the Raman spectra of *S. violaceoruber* at 1342 and 1 586 cm^−1^ are both amino acid signals, which might correspond to l-glutamate and l-phenylalanine, respectively [61, 62]. Moreover, although *Streptomyces* spores are known to contain trehalose, no signal for this chemical was found [63, 64]. Many other organisms aside from *Streptomyces* are known to accumulate this sugar, as it is known to aid in the resistance to desiccation [65, 66]. The absence of the trehalose signal in our study could be due to background noise originating from the pigments produced by *Streptomyces *. In contrast, the two peaks (1120 and 1149 cm^−1^) of *M. xanthus *’s spore spectra that were significantly different from the vegetative cell spectra are part of the trehalose Raman signal [61]. As in the case of *Streptomyces, Myxococcus*’ spores are known to accumulate this compound [63, 67, 68]. Similarly, trehalose is also known to be accumulated in the cysts of *Azotobacter* [21, 69], and accordingly the trehalose peak at 830 cm^−1^ [61] is one of the Raman markers in *A. chroococcum *’s spore spectra. Future studies could be focused on the evaluation of trehalose as a marker for the response of microbial communities to desiccation, such as in conditions of soil drought.

In contrast to *S. violaceoruber *, *M. xanthus* and *A. chroococcum,* the vegetative and spore spectra of *A. cylindrica* did not show unique signals that could be used for the identification of either cell type. The only difference among the *A. cylindrica *’s spectra was the increase in intensity in the spore’s spectra in comparison to the vegetative cell and the disappearance of the peak at 2295 cm^−1^ in the spore. Therefore, in the case of akinetes, chemical signatures appear not to be an option for their study in complex microbial communities. Other physical properties, such as lack of buoyancy, could be evaluated in the future.

In the case of *B. subtilis *’s spores (and the other endospore-formers tested here), a high concentration of CaDPA was observed. The signal of CaDPA was not found in the vegetative cell’s spectra. This chemical (i.e. CaDPA) is a well-known component of endospores that has been shown to have an important role in heat resistance and germination of endospores [9, 58, 70, 71]. This striking chemical difference between vegetative cells and endospores was exploited here as a proof of concept of the feasibility of using individual spore-specific Raman markers to isolate endospores from environmental samples.

In previous studies, detection of CaDPA using Raman microspectroscopy has been used to assess the presence of endospores in pure cultures and powder samples [72, 73]. CaDPA has also been used extensively in the past for the quantification and detection of endospores. For instance, quantification of endospores has often been done through the detection of CaDPA using high performance liquid chromatography (HPLC) or Tb-DPA (i.e. DPA chelated with Tb) photoluminescence, after its release by autoclaving, bead beating, or by inducing germination [74–76]. However, these methods infer abundance assuming a standard concentration of CaDPA per spore, even though this concentration is known to vary between strains [75, 77].

Another molecular marker used to identify and quantify endospores is the gene encoding the regulatory protein Spo0A [78]. However, the quantification of *spoA* requires specialized DNA extraction methods to break the highly lysis-resistant endospores [79–81].

All of the methods indicated above allow for detection and quantification, but not isolation. The markers developed in our study offer a nondestructive approach that enables not only the identification of single-cell endospores, but also further isolation or single-cell molecular studies when combined with Raman-activated microbial cell sorting (RACS [45, 82, 83]). This type of platform combines single-cell Raman microspectroscopy with microfluidics to sort cells according to a previously established threshold. This approach would provide a wealth of information in environments in which endospores are known to be highly prevalent, such as the human gut microbiome [84–86] or the deep biosphere [74, 87, 88]. This can also be an important tool in environmental forecasting, for example, for studying the prevalence of antibiotic resistance genes associated with endospores in sediments [89, 90].

In conclusion, our results on the morphological and chemical composition of sporulating bacterial models show the absence of chemical or morphological universal markers for spores. The information available on genetic markers also corroborates the non-universality of sporulation. Indeed, individual sporulation genetic markers have been found for Firmicutes [91, 92], but in the case of Actinobacteria, Cyanobacteria, *Myxococcus* and *Azotobacter *, the high genetic variation among species [93, 94] or the complexity of the sporulation process [21, 23] has made the identification of general molecular markers for exospores, akinetes, myxospores and/or cysts impossible. We also presented the validation of an endospore-specific Raman marker as a proof of concept of the feasibility of identifying individual spore markers for other spore types (i.e. exospores, akinetes, myxospores and cysts). These markers could open the door to future investigations on the isolation and characterization of the underexplored bacterial seed bank.

## Supplementary Data

Supplementary material 1
