# Supplementary material for: Multiple roads lead to Rome: unique morphology and chemistry of endospores, exospores, myxospores, cysts and akinetes in bacteria
Source: Microbiology (Reading). 2023 Feb 20;169(2):001299. doi: 10.1099/mic.0.001299 (PMC10197873; doi:10.1099/mic.0.001299)
Supplement: Supplementary material 1 [file mic-169-1299-s002.pdf]

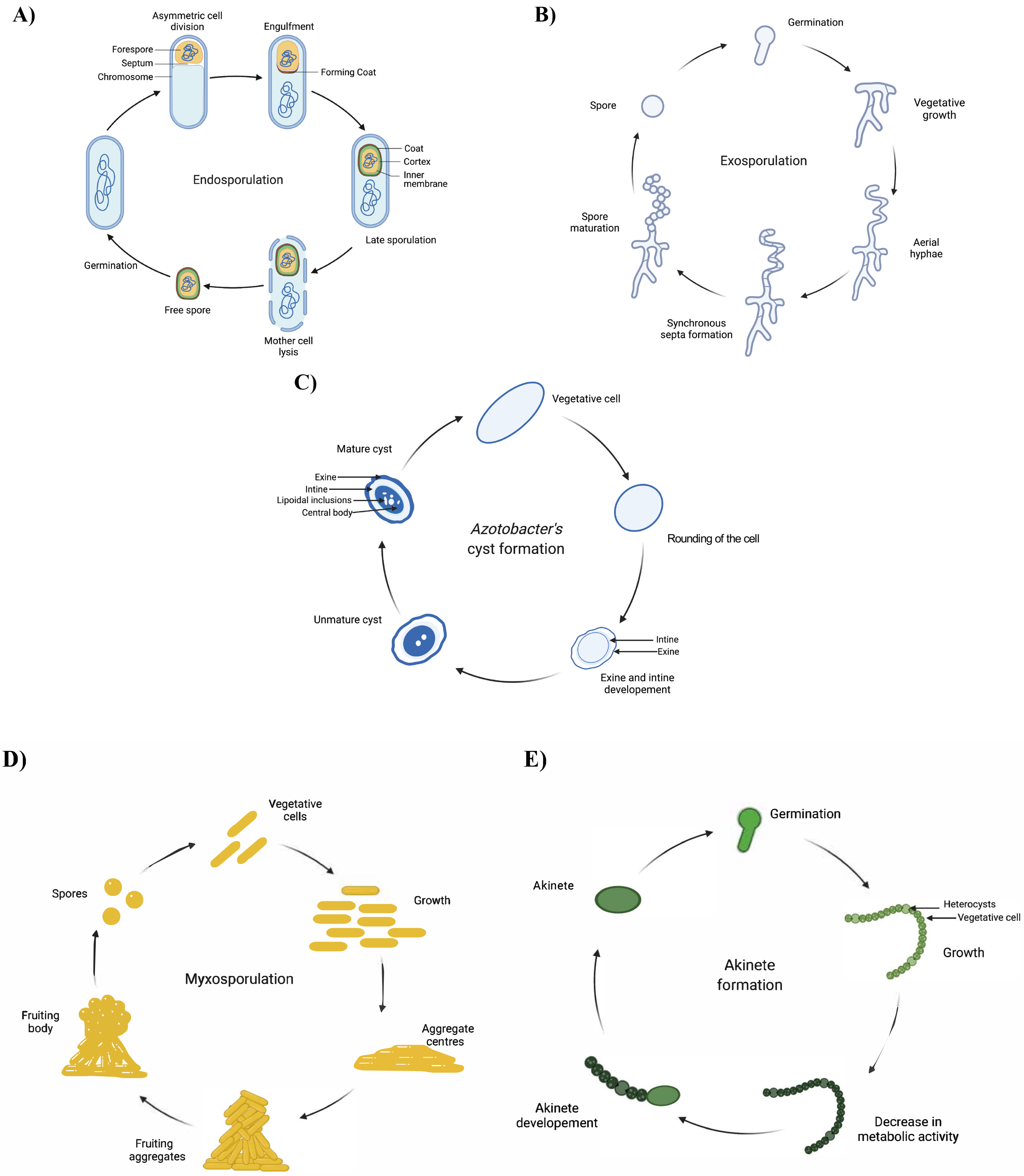

Supplementary Figure 1. Diagram of the formation of A) Endospores produced by some members of the Firmicutes phylum, B) Exospores produced by some members of the Actinobacteria phylum, C) Cyst produced by some members of the Azotobacteracea order within Proteobacteria. E) Myxospores produced by some members of the Myxococcales order within Proteobacteria, B) Akinetes produced by some members of the Cyanobacteria phylum.

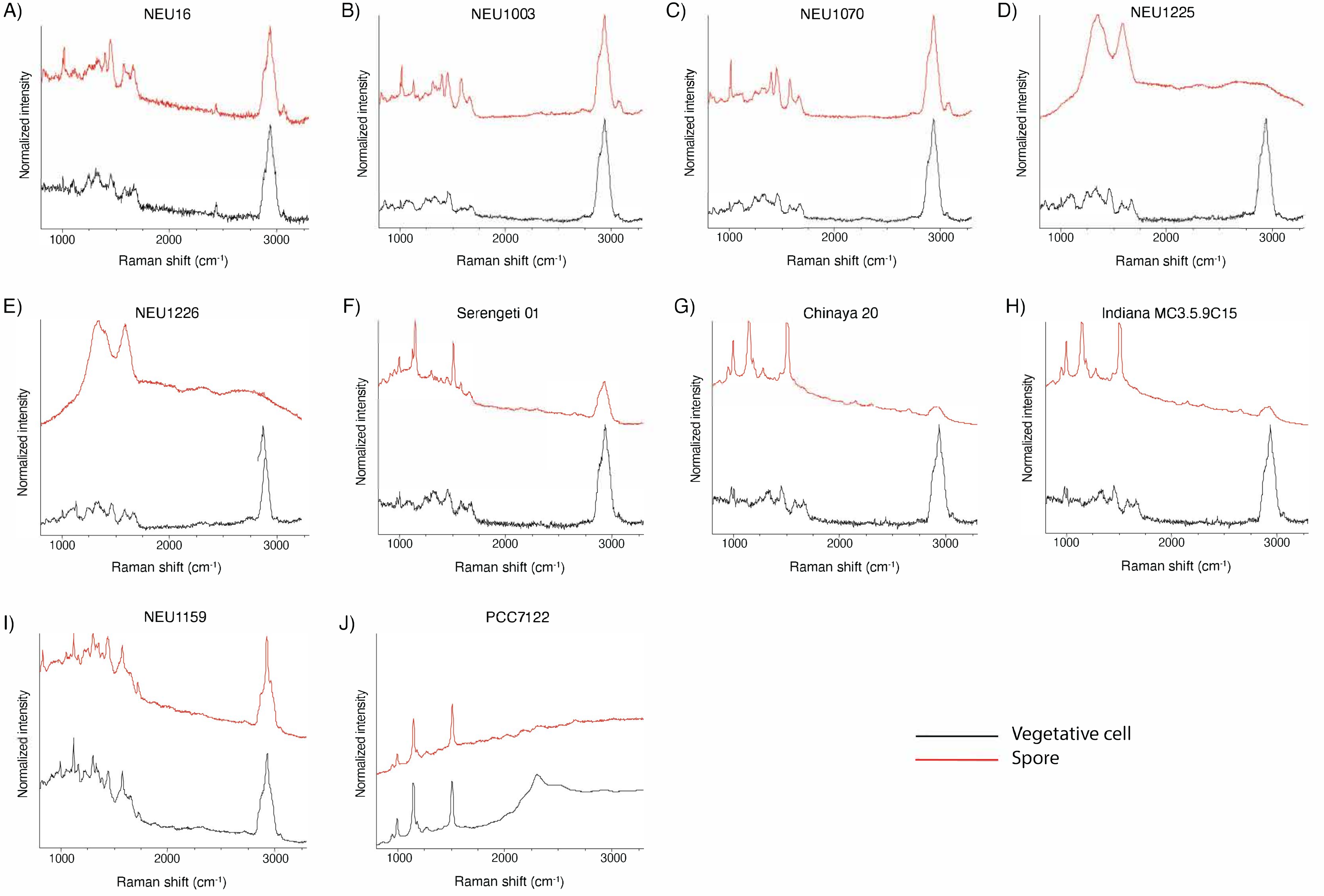

Supplementary Figure 2. Representative Raman spectra of a vegetative cell (black) or spore (red) of **A)** *Bacillus subtilis*, **B)** *Bacillus sphaericus*, **C)** *Bacillus thuringiensis*, **D)** *Streptomyces violaceoruber*, **E)** *Streptomyces avermitilis*, **F-H)** *Myxococcus xanthus*, **I)** *Azotobacter chroococcum*, **J)** *Anabaena cylindrica*. For each sample 15 to 20 individual cells were measured, and a representative Raman spectrum normalized with respect to the maximum and minimum intensities (I - Imin / Imax - Imin) is displayed. No further data processing (smoothing or baseline subtraction) was conducted.
